# Sustained injection of miR-499-5p alters the gastrocnemius muscle metabolome in broiler chickens

**DOI:** 10.5194/aab-65-275-2022

**Published:** 2022-08-03

**Authors:** Chuwen Chen, Jie Li, Zhixiong Li

**Affiliations:** 1 Key Laboratory of Qinghai-Tibetan Plateau Animal Genetic Resource Reservation and Utilization, Ministry of Education, Southwest Minzu University, Chengdu, 610041, China; 2 College of Animal & Veterinary Sciences, Southwest Minzu University, Chengdu, 610041, China

## Abstract

To investigate the effects of miR-499-5p on muscle metabolism in broiler chickens, eight broiler chicks were assigned to the control group and eight to the treatment group, and then we monitored the effects using metabolomics. Chicks were fed basal diets without or with miR-499-5p delivery. Gastrocnemius muscle samples were collected and analyzed by ultrahigh-performance liquid chromatography–tandem mass spectrometry. The results showed that miR-499-5p injection altered the concentrations of a variety of metabolites in the gastrocnemius muscle. Thereby, a total of 46 metabolites were identified at higher ( P<0.05 ) concentrations and 30 metabolites were identified at lower ( P<0.05 ) concentrations in the treatment group compared with the control group. These metabolites were primarily involved with the regulation of lipid and carbohydrate metabolism. Further metabolic pathway analysis revealed that fructose and mannose metabolism, galactose metabolism, inositol phosphate metabolism, and terpenoid backbone biosynthesis were the most critical pathway which may partially interpret the effects of miR-499-5p. To our knowledge, this research is the first report of metabolic signatures and related metabolic pathways in the skeletal muscle for miR-499-5p injection and provides new insight into the effect of miRNA on growth performance.

## 1 Introduction

Broilers have been one of the most important sources of meat for humans, and their productivity has been substantially improved by molecular-marker-assisted selection. As the main livestock product of broilers, skeletal muscle constitutes approximately 40 % of body mass. Apart from extrinsic regulators of myogenesis, several levels of intrinsic complexity arise from hierarchical interactions between transcriptional regulators and regulatory RNAs. Several studies showed that post-transcriptional regulation of miRNA has a significant effect on skeletal muscle development (Luo et al., 2013; Horak et al., 2016). However, the precise metabolic mechanisms are still poorly understood.

MicroRNAs (miRNAs) are post-transcriptional regulators that bind to the target messenger RNA's (mRNA) 3 ${}^{\prime }$ -untranslated region (3 ${}^{\prime }$ -UTR), usually resulting in translational repression in mammals (Bartel, 2009; Gladka et al., 2012). Many miRNAs seem to be expressed in a muscle-specific manner and are as a group often referred to as myogenic miRNAs (myomiRs) (Mccarthy et al., 2009). The expression of myomiRs is dramatically increased during myogenesis (Chen et al., 2006). MyomiRs have been shown to play critical roles in many aspects of muscle function, including muscle development, satellite cell activity, and muscle fiber specification (Xu et al., 2018; Liu et al., 2016; Cheung et al., 2012).

As a myomiR, miR-499-5p is highly expressed in cardiac and skeletal muscle and is encoded by myosin heavy chain 7b (MyHC7b), which is a member of the MyHC family (Van Rooij et al., 2009). It has been identified to be an important regulator of muscle fiber type transition (Bhuiyan et al., 2013; X. Wang et al., 2011b). It was reported that miR-499-5p played a dominant role in the specification of muscle fiber identity by activating slow and repressing fast myofiber genes (Van Rooij et al., 2009). Several transcriptional repressors such as *Sox6* and *Pur* β , which have been determined to inhibit MyHC7b transcriptional activity, were identified as miR-499-5p target genes (Van Rooij et al., 2009; X. Wang et al., 2011b, 2017). However, the underlying physiological and metabolic mechanisms in regulation of skeletal muscle by miR-499-5p remained largely unknown.

Metabolomics provide a powerful platform for identifying small molecular metabolites in biological samples (biofluids or tissues) using high-throughput approaches. The identification and integrative analysis of these metabolites can facilitate the characterization of metabolism at the molecular and cellular levels under a given set of physiological conditions (Patti et al., 2012; Tan et al., 2021; Wen et al., 2020; Liu et al., 2021). In the present study, we used ultrahigh-performance liquid chromatography–tandem mass spectrometry (UHPLC-MS/MS) to identify the metabolic phenotype variation associated with overexpression of miR-499-5p. The results are of great significance for the metabolic mechanism of miR-499-5p in skeletal muscle.

## 2 Materials and methods

### 2.1 Animals and experimental design

A total of 16 14 d old male broilers were randomly divided into a treatment group (TG) and control group (CG), with eight chicks in each group. All broilers were fed basal diets, housed in wired cages, offered free access to feed and water, with a lighting schedule of 20 h light and 4 h dark. AgomiRs of miR-499-5p, synthesized from Ribobio, were chemically engineered from cholesterol-modified oligonucleotides to mimic miRNA expression and injected intramuscularly into gastrocnemius muscle at a dose of 5 nmol. A scramble miRNA agomiR was used as the negative control. The injections were repeated every 72 h and given five times to ensure efficacy. Gastrocnemius muscles were taken from each bird a week after the last injection; all fresh tissue samples were washed briefly with phosphate-buffered saline (PBS) and divided into divided into three parts. Two parts for analysis by UHPLC-MS/MS and qPCR were immediately frozen in liquid nitrogen and stored at - 80 ${}^{\circ }$ , and the other part was fixed in 4 % paraformaldehyde and embedded in paraffin for histological observation.

### 2.2 Quantitative analysis of miR-499-5p

Stem-loop quantitative real-time polymerase chain reaction (stem-loop qPCR) was used to analyze the expression of miR-499-5p (Chen et al., 2005). The stem-loop qPCR was performed in the Bio-Rad CFX96 real-time PCR detection system using the SYBR Green PCR kit (Takara, Japan). 5S rRNA was used as the reference gene in the stem-loop qPCR detection of miR-499-5p, and all reactions were run in triplicate. The primers for the qPCR are shown in Table 1.

### 2.3 Histological examination of the gastrocnemius muscle

The histological characteristics in the gastrocnemius were evaluated by haematoxylin and eosin (H&E) staining. Paraffin sections were mounted on slides for hematoxylin and eosin staining. Histological characteristics of the chicken skeletal muscle were observed using a BA210 digital microscope (Motic) and Images Advanced Software (Motic). All 16 chicks in two groups were evaluated in the experiment. Five images for each sample were collected for the statistics of muscle fiber diameter.

### 2.4 Statistical analysis

Data were expressed in mean ± SD. Statistical analysis was carried out using one-way analysis of variance (ANOVA) with the SPSS software (version 26.0). The significant value between groups was set at P<0.05 .

### 2.5 Metabolite extraction

The 16 samples of gastrocnemius muscle were individually grounded with liquid nitrogen, and the homogenate was resuspended with prechilled 80 % methanol and 0.1 % formic acid by vortexing well. The samples were incubated on ice for 5 min and then were centrifuged at 15 000 rpm and 4 ${}^{\circ }$ C for 5 min. Some of the supernatant was diluted to a final concentration containing 60 % methanol by LC-MS-grade water. The samples were subsequently transferred to a fresh Eppendorf tube with a 0.22 µ m filter and then were centrifuged at 15 000 g and 4 ${}^{\circ }$ C for 10 min. Finally, the filtrate was injected into the LC-MS/MS system analysis. Quality control (QC) samples were also prepared by mixing equal volumes of each sample; the samples were aliquoted for analysis prior to sample preparation. The QC samples were used to monitor deviations of the analytical results from the pooled mixtures and compare to the errors caused by the analytical instrument itself.

**Figure 1:**
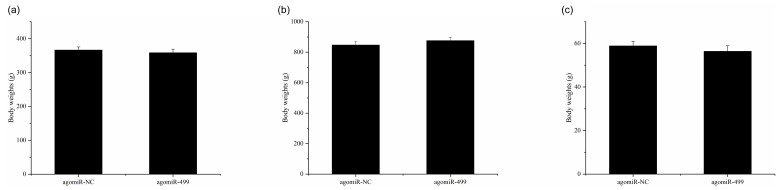
The body weights of chicks in the control group (CG) and treatment group (TG). **(a)** The body weights of chicks in the two groups at day 1. **(b)** The body weights of chicks in the two groups at day 18. **(c)** The leg muscle weights of chicks in the two groups at day 18. The agomiR-NC and agomiR-499 represent the control and treatment groups, respectively.

### 2.6 Metabolomic analysis of muscle samples

LC-MS/MS analyses were performed using a Vanquish UHPLC system (Thermo Fisher) coupled with an Orbitrap Q Exactive HF-X mass spectrometer (Thermo Fisher). Samples were injected onto a Hyperil Gold column (100 × 2.1 mm, 1.9 µ m) using a 16 min linear gradient at a flow rate of 0.2 mL min ${}^{-1}$ . The eluents for the positive polarity mode were eluent A (0.1 % FA in water) and eluent B (methanol). The eluents for the negative polarity mode were eluent A (5 mM ammonium acetate, pH 9.0) and eluent B (methanol). The solvent gradient was set as follows: 2 % B, 1.5 min; 2 %–100 % B, 12.0 min; 100 % B, 14.0 min; 100 %–2 % B, 14.1 min; 2 % B, 16 min. The Q Exactive HF-X mass spectrometer was operated in positive–negative polarity mode with a spray voltage of 3.2 kV, capillary temperature of 320 ${}^{\circ }$ C, sheath gas flow rate of 35 arb, and auxiliary gas flow rate of 10 arb.

**Table 1:** Increased metabolites for the agomiR-499-treated group compared with the control.

Metabolite name	Molecular formula	Retention time	FC ${}^{a}$	P value	VIP ${}^{b}$
Joro toxin	C ${}_{27}$ H ${}_{47}$ N ${}_{7}$ O ${}_{6}$	14.54	2.72	<0.001	3.08
Palmatine	C ${}_{21}$ H ${}_{22}$ N O ${}_{4}$	13.93	1.77	<0.001	1.82
O-heptanoylcarnitine	C ${}_{14}$ H ${}_{27}$ N O ${}_{4}$	9.88	2.59	<0.001	3.07
Stearoylcarnitine	C ${}_{25}$ H ${}_{49}$ N O ${}_{4}$	15.23	2.04	<0.001	2.24
4,6-Henicosanedione	C ${}_{21}$ H ${}_{40}$ O ${}_{2}$	15.44	14.63	<0.001	7.37
Valeric acid	C ${}_{5}$ H ${}_{10}$ O ${}_{2}$	6.68	1.93	0.001	2.03
Methyl 9-octadecenoate	C ${}_{19}$ H ${}_{36}$ O ${}_{2}$	15.22	5.48	0.001	4.93
Reduced vitamin K	C ${}_{31}$ H ${}_{46}$ O ${}_{2}$	14.00	1.92	0.001	2.02
7-Alpha-hydroxy-3-oxochol-4-en-24-oic acid	C ${}_{24}$ H ${}_{36}$ O ${}_{4}$	13.54	2.74	0.001	2.98
5-O-mycaminosyprotylonolide	C ${}_{31}$ H ${}_{53}$ N O ${}_{8}$	14.85	2.57	0.001	2.86
(2Z)-4-(Octadecyloxy)-4-oxo-2-butenoic acid	C ${}_{22}$ H ${}_{40}$ O ${}_{4}$	14.00	2.01	0.002	2.17
N-Stearoyl-L-tyrosine	C ${}_{27}$ H ${}_{45}$ N O ${}_{4}$	13.54	2.24	0.002	2.42
Oleoyl tyrosine	C ${}_{27}$ H ${}_{43}$ N O ${}_{4}$	13.33	3.26	0.002	3.58
*cis*-2-Carboxycyclohexyl-acetic acid	C ${}_{9}$ H ${}_{14}$ O ${}_{4}$	7.14	2.57	0.002	2.75
Linoleyl carnitine	C ${}_{25}$ H ${}_{45}$ N O ${}_{4}$	13.56	2.11	0.002	2.17
Hydroprene	C ${}_{17}$ H ${}_{30}$ O ${}_{2}$	14.12	6.25	0.002	4.89
16,16-Dimethyl prostaglandin A1	C ${}_{22}$ H ${}_{36}$ O ${}_{4}$	13.55	3.16	0.002	3.29
Promolate	C ${}_{16}$ H ${}_{23}$ N O ${}_{4}$	8.58	2.96	0.003	3.56
Propionylcarnitine	C ${}_{10}$ H ${}_{19}$ N O ${}_{4}$	2.07	2.81	0.003	3.37
Benzamide	C ${}_{7}$ H ${}_{7}$ N O	13.79	1.65	0.003	1.47
Pregnane-3,3-diol	C ${}_{21}$ H ${}_{36}$ O ${}_{2}$	14.99	5.38	0.004	5.07
Decylubiquinone	C ${}_{19}$ H ${}_{30}$ O ${}_{4}$	13.80	1.68	0.004	1.51
Methyl stearate	C ${}_{19}$ H ${}_{38}$ O ${}_{2}$	15.34	2.80	0.005	2.81
Cassaidine	C ${}_{24}$ H ${}_{41}$ N O ${}_{4}$	12.99	6.80	0.005	4.76
Iminoctadine	C ${}_{18}$ H ${}_{41}$ N ${}_{7}$	14.72	2.46	0.006	2.63
DO0750000	C ${}_{15}$ H ${}_{24}$ O ${}_{2}$	13.97	4.53	0.006	5.22
Palmitoylcarnitine	C ${}_{23}$ H ${}_{45}$ N O ${}_{4}$	13.69	1.84	0.007	1.81
Glyceraldehyde 3-phosphate	C ${}_{3}$ H ${}_{7}$ O ${}_{6}$ P	1.18	2.02	0.008	2.18
11-Deoxy prostaglandin F1 α	C ${}_{20}$ H ${}_{36}$ O ${}_{4}$	13.69	2.53	0.011	2.65
*trans*-2-Tetradecenoylcarnitine	C ${}_{21}$ H ${}_{39}$ N O ${}_{4}$	12.98	1.80	0.012	1.73
O-oleoylcarnitine	C ${}_{25}$ H ${}_{47}$ N O ${}_{4}$	13.77	2.07	0.012	2.02
Xanthine	C ${}_{5}$ H ${}_{4}$ N ${}_{4}$ O ${}_{2}$	1.72	1.71	0.013	1.90
Erucic acid	C ${}_{22}$ H ${}_{42}$ O ${}_{2}$	15.67	1.77	0.015	1.72
8,9-DiHETrE	C ${}_{20}$ H ${}_{34}$ O ${}_{4}$	13.42	2.20	0.019	2.57
Clominorex	C ${}_{9}$ H ${}_{9}$ Cl N ${}_{2}$ O	4.99	1.51	0.024	1.35
1-Hexadecyl-sn-glycerol 3-phosphate	C ${}_{19}$ H ${}_{41}$ O ${}_{6}$ P	14.60	1.47	0.026	1.15
O-pentadecanoylcarnitine	C ${}_{22}$ H ${}_{43}$ N O ${}_{4}$	13.50	1.86	0.029	1.79
5-Alpha-cholane-3alpha,7alpha,12alpha,24-tetrol	C ${}_{24}$ H ${}_{42}$ O ${}_{4}$	14.07	1.72	0.030	1.69
10-Deoxymethymycin	C ${}_{25}$ H ${}_{43}$ N O ${}_{6}$	13.61	2.02	0.031	2.30
(2E)-Hexadecenoylcarnitine	C ${}_{23}$ H ${}_{43}$ N O ${}_{4}$	13.42	1.63	0.034	1.45
16-Acetoxy-17-methoxy-17-oxokauran-18-oic acid	C ${}_{23}$ H ${}_{34}$ O ${}_{6}$	11.71	1.77	0.034	2.02
( + /-)-Camphoric acid	C ${}_{10}$ H ${}_{16}$ O ${}_{4}$	8.80	1.59	0.034	1.44
PD-128042	C ${}_{23}$ H ${}_{39}$ N O ${}_{4}$	12.86	2.25	0.041	2.58
Lersivirine	C ${}_{17}$ H ${}_{18}$ N ${}_{4}$ O ${}_{2}$	15.03	1.56	0.042	1.28
O-heptadecanoylcarnitine	C ${}_{24}$ H ${}_{47}$ N O ${}_{4}$	13.81	2.27	0.043	2.31
Hexanoylcarnitine	C ${}_{13}$ H ${}_{25}$ N O ${}_{4}$	8.80	1.60	0.045	1.42

${}^{a}$ FC: fold change for the treatment group to control. ${}^{b}$ VIP: variable importance in the projection.

### 2.7 Data processing and analysis

The raw data files generated by UHPLC-MS/MS were processed using Compound Discoverer 3.0 (CD 3.0, Thermo Fisher) to perform peak alignment, peak picking, and quantitation for each metabolite. The main parameters were set as follows: retention time tolerance, 0.2 min; actual mass tolerance, 5 ppm; signal intensity tolerance, 30 %; signal / noise ratio, 3; and minimum intensity, 100 000. After that, peak intensities were normalized to the total spectral intensity. The normalized data were used to predict the molecular formula based on additive ions, molecular ion peaks, and fragment ions. And then peaks were matched with the mzCloud (https://www.mzcloud.org/, last access: 25 October 2020) and ChemSpider (http://www.chemspider.com/, last access: 25 October 2020) databases to obtain the accurate qualitative and relative quantitative results.

For multivariate statistical analysis, both principal component analysis (PCA) and orthogonal projections to latent structures discriminant analyses (OPLS-DA) were performed to visualize the differences between groups. PCA and OPLS-DA were both performed using the SIMCA-P software (version 13.0). PCA was firstly employed to visualize the sample clustering, trends, and outliers among the observations. Then OPLS-DA was performed to highlight the difference between groups. The OPLS-DA model was validated by 200 random permutations tests for avoiding overfitting. Afterward, loading plots were constructed, which showed the contribution of variables to the difference between the two groups. It also showed the important variables which were situated far from the origin, but the loading plot is complex because of many variables. To refine this analysis, the first principal component of variable importance in the projection (VIP) was obtained through OPLS-DA. Metabolites were annotated and identified based on accurate mass and MS information by searching through the database. Metabolites were finally verified by comparing retention times and fragmentation patterns with standards. The fold change (FC) value of each metabolite was calculated by comparing mean peak values obtained from the TG to that from the CG. Differential metabolites were selected based on the basis of the VIP value ( > 1.0), FC value (FC > 1.2 or FC < 0.833), and Student's t test ( P<0.05 ). Pearson's product-moment correlation was performed to calculate the correlation. Corresponding P values and false discovery rates (FDRs) of each correlation were also calculated using the “cor. test function” in R software. Differential metabolites were further mapped onto general biochemical pathways according to annotation in the Kyoto Encyclopedia of Genes and Genomes (KEGG).

## 3 Results

### 3.1 Effect of miR-499-5p overexpression on body weight and muscle fiber diameter

**Figure 2:**
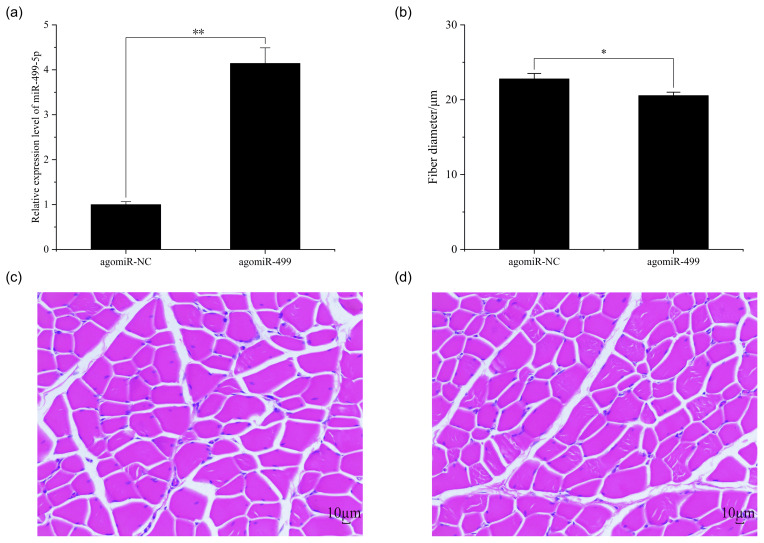
The muscle fiber in the control group (CG) and treatment group (TG). **(a)** The expression of miR-499-5p in the TG and CG. **(b)** The diameter of muscle fiber in the CG and TG. **(c)** HE staining of gastrocnemius in the CG. **(d)** HE staining of gastrocnemius in the TG. Statistical significance is indicated by ${}^{*}$ P<0.05 . The agomiR-NC and agomiR-499 represent the control and treatment groups, respectively.

The body weights of broilers in the two groups were monitored at the beginning and end of the experiment period. As shown in Fig. 1, the body weights of broilers in the two groups at day 1 and day 18 were presented. There were no significant differences in the initial body weights of each group on day 1 (Fig. 1a). After intramuscular injection of agomiRs of miR-499-5p and negative control five times, there were still no significant differences in the body and leg muscle weights on day 18 (Fig. 1b and c). The expression of miR-499-5p was much higher in the TG compared to that in the CG (Fig. 2a, P<0.01 ). Different from the broilers in the CG, a dramatic decrease in the diameter of muscle fiber can be found in the TG in (Fig. 2b, c, and d, P<0.05 ).

### 3.2 Characterization of LC-MS/MS data

**Figure 3:**
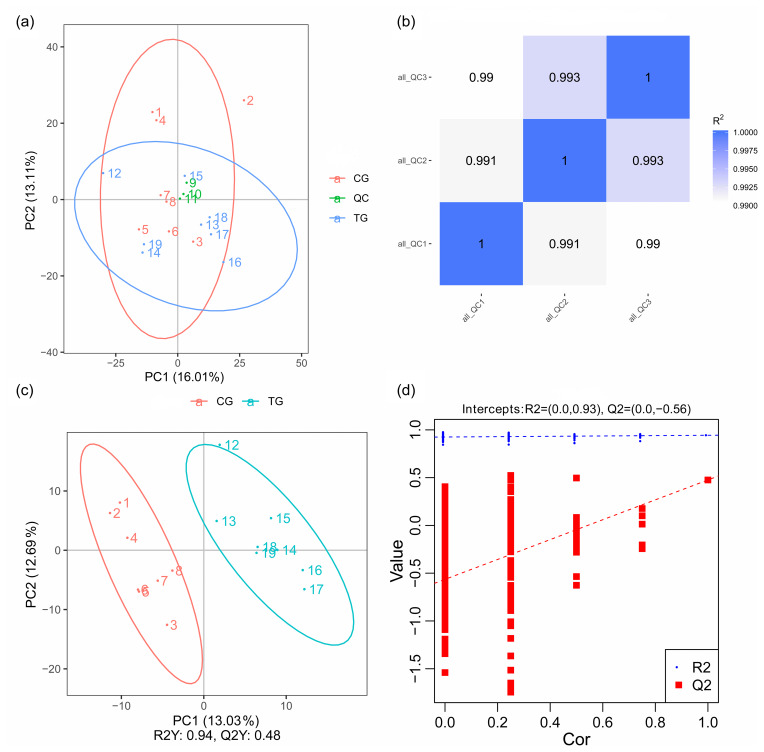
PCA and OPLS-DA score plots. **(a)** PCA score plots for consecutively analyzed quality control (QC) samples. **(b)** The Pearson correlation coefficient of three QC samples. **(c)** OPLS-DA score plots discriminating the control group (CG) and treatment group (TG). **(d)** Permutation test for the OPLS-DA model C.

PCA mainly shows the distribution of the original data, which reduces the dimensionality of data and summarizes the similarities and differences between multiple MS spectra using score plots. In the present study, PCA was performed, and the result revealed that most of the muscle samples in the score plots were inside the 95 % Hotelling $T^{2}$ ellipse (Fig. 3a). The correlation of three QC samples was calculated by the “Pearson” correlation coefficient, and the results showed that the correlation of all the QC samples exceeds 99 % (Fig. 3b). As a supervised multivariate classification tool, the OPLS-DA model was constructed following PCA for obtaining an improved separation and gaining a better understanding of the variables responsible for the classification. As shown in Fig. 3c, all the samples in the OPLS-DA score plots were within the 95 % Hotelling $T^{2}$ ellipse. The $R^{2}Y$ value of the OPLS-DA model that represents the explained variance was 0.94. The cross-validation indicated the excellent predictive ability of this model, with a relatively high $Q^{2}$ value of 0.48. The OPLS-DA model exhibited a clear separation between the TG and CG. Furthermore, a permutation test was applied to assess the robustness and predictive ability of the OPLS-DA model (Fig. 3d). The corresponding $R^{2}Y$ and $Q^{2}$ intercept values were 0.93 and - 0.56, respectively, indicating satisfactory effectiveness of the OPLS-DA model.

### 3.3 Differential metabolites in gastrocnemius

**Figure 4:**
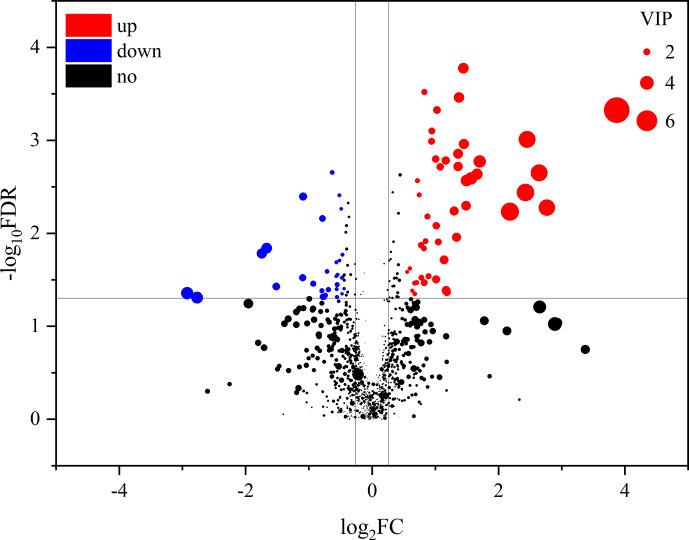
Volcano plots of metabolites in muscle between the control group and treatment group. Each dot represents a metabolite. The larger dots indicate higher variable importance in the projection (VIP) values. The abscissa and ordinate represent the fold change and P value of metabolites, respectively. The increased and decreased ( P<0.05 ) metabolites in the treatment group (TG) are represented by the red and blue dots, respectively, and the black dots represent the unchanged metabolites ( P>0.05 ) between the two groups.

An obvious separation can be observed between the treatment and control group in the OPLS-DA model, indicating that there was a significant difference in the metabolome of the gastrocnemius of the two groups. We determined those differentially expressed metabolites that played important roles in separating the treatment and control groups. Differential metabolites between the two groups were selected when the P values of the Student's t test were less than 0.05 and the VIP values were more than 1.0. The profile of differential metabolites between the TG and CG was visualized by a volcano plot (Fig. 4). A total of 76 differential metabolites were testified using MS/MS analysis (Tables 1 and 2) based on these criteria. Of the identified metabolites, 46 metabolites were found at higher levels, whereas 30 metabolites were found at lower levels in the TG compared with the CG. These metabolites are primarily involved in the metabolic processes of carbohydrates, nucleotides, and lipids. On the basis of the FC value, several metabolites were determined including 7-alpha-hydroxy-3-oxochol-4-en-24-oic acid (FC = 2.74), 5-alpha-cholane-3-alpha,7-alpha,12-alpha,24-tetrol (FC = 1.72), O-heptanoylcarnitine (FC = 2.59), stearoylcarnitine (FC = 2.04), linoleyl carnitine (FC = 2.11), propionylcarnitine, (FC = 2.81), palmitoylcarnitine (FC = 1.84), *trans*-2-tetradecenoylcarnitine (FC = 1.80), O-oleoylcarnitine (FC = 2.07), O-pentadecanoylcarnitine (FC = 1.86), (2E)-hexadecenoylcarnitine (FC = 1.63), O-heptadecanoylcarnitine (FC = 2.27), and hexanoylcarnitine (FC = 1.60) along with taurochenodeoxycholic acid (FC = 0.13), palmitelaidic acid (FC = 0.68), phloionolic acid (FC = 0.61), and lauric acid (FC = 0.31).

### 3.4 Metabolic pathway enrichment analysis

The differential metabolites detected in gastrocnemius in the present study pinpointed the involved pathways. As shown in Table 3, a total of 13 pathways were obtained when the differential metabolites between the two groups were imported into the KEGG database. These metabolites were distributed among the metabolic pathways of fructose and mannose metabolism, galactose metabolism, inositol phosphate metabolism, terpenoid backbone biosynthesis, glycolysis and gluconeogenesis, caffeine metabolism, vitamin B6 metabolism, primary bile acid biosynthesis, thiamine metabolism, pentose phosphate pathway, fatty acid biosynthesis, biosynthesis of unsaturated fatty acids, and purine metabolism. Among them, fructose and mannose metabolism, galactose metabolism, inositol phosphate metabolism, and terpenoid backbone biosynthesis exhibited significant differences ( P<0.05 ), so these four metabolic pathways were thus characterized as the significantly relevant pathways associated with the metabolic changes in chicks due to miR-499-5p injection.

**Table 2:** Decreased metabolites for the agomiR-499-treated group compared with the control.

Metabolite name	Molecular formula	Retention time	FC ${}^{a}$	P value	VIP ${}^{b}$
Cortisol, 9-fluoro-16. alpha.-hydroxy-	C ${}_{21}$ H ${}_{29}$ F O ${}_{6}$	12.74	0.65	0.002	1.41
Diosgenin	C ${}_{27}$ H ${}_{42}$ O ${}_{3}$	15.11	0.70	0.004	1.15
Triamciolone diacetate	C ${}_{25}$ H ${}_{31}$ F O ${}_{8}$	14.75	0.47	0.004	2.36
Geranylacetone	C ${}_{13}$ H ${}_{22}$ O	13.73	0.71	0.005	1.09
KJ9800000	C ${}_{18}$ H ${}_{39}$ O ${}_{7}$ P	15.31	0.58	0.007	1.94
Lauric acid	C ${}_{12}$ H ${}_{24}$ O ${}_{2}$	13.29	0.31	0.014	3.15
Dihydroconiferyl alcohol glucoside	C ${}_{16}$ H ${}_{24}$ O ${}_{8}$	8.00	0.30	0.016	2.92
3-Dehydro-2-deoxyecdysone	C ${}_{27}$ H ${}_{42}$ O ${}_{5}$	15.07	0.72	0.017	1.13
Ibufenac	C ${}_{12}$ H ${}_{16}$ O ${}_{2}$	12.14	0.70	0.020	1.09
Palmitelaidic acid	C ${}_{16}$ H ${}_{30}$ O ${}_{2}$	13.25	0.68	0.020	1.19
Phloionolic acid	C ${}_{18}$ H ${}_{36}$ O ${}_{5}$	12.38	0.61	0.026	1.36
Spiro[3H-indole-3,5 ${}^{\prime }$ (4 ${}^{\prime }$ H)-thiazol]-2-ol, 2 ${}^{\prime }$ -(methylthio)-	C ${}_{11}$ H ${}_{10}$ N ${}_{2}$ O S ${}_{2}$	1.25	0.69	0.028	1.16
MFCD00010043	C ${}_{16}$ H ${}_{10}$ S	1.25	0.68	0.029	1.13
(17-beta)-4-(Acetylsulfanyl)-3-oxoandrost-4-en-17-yl propionate	C ${}_{24}$ H ${}_{34}$ O ${}_{4}$ S	9.65	0.47	0.030	2.07
Quinagolide	C ${}_{20}$ H ${}_{33}$ N ${}_{3}$ O ${}_{3}$ S	15.55	0.72	0.030	1.09
Buclizine	C ${}_{28}$ H ${}_{33}$ Cl N ${}_{2}$	10.15	0.52	0.035	1.75
Sulbutiamine	C ${}_{32}$ H ${}_{46}$ N ${}_{8}$ O ${}_{6}$ S ${}_{2}$	13.99	0.68	0.036	1.49
(2S)-2-Piperazinecarboxamide	C ${}_{5}$ H ${}_{11}$ N ${}_{3}$ O	1.29	0.68	0.036	1.15
Probucol	C ${}_{31}$ H ${}_{48}$ O ${}_{2}$ S ${}_{2}$	13.45	0.35	0.037	2.28
( - )-Prostaglandin E1	C ${}_{20}$ H ${}_{34}$ O ${}_{5}$	12.50	0.74	0.040	1.01
NK7755000	C ${}_{11}$ H ${}_{11}$ Cl N ${}_{2}$ O ${}_{2}$	0.10	0.68	0.040	1.14
Toborinone	C ${}_{21}$ H ${}_{24}$ N ${}_{2}$ O ${}_{5}$	13.23	0.62	0.040	1.54
Bardoxolone methyl	C ${}_{32}$ H ${}_{43}$ N O ${}_{4}$	14.92	0.58	0.042	1.44
Taurochenodeoxycholic acid	C ${}_{26}$ H ${}_{45}$ N O ${}_{6}$ S	12.82	0.13	0.044	3.60
1-Palmitoyl-2-(5-keto-6-octendioyl)-sn-glycero-3-phosphatidylcholine	C ${}_{32}$ H ${}_{58}$ N O ${}_{11}$ P	14.92	0.72	0.045	1.02
Persin	C ${}_{23}$ H ${}_{40}$ O ${}_{4}$	14.96	0.60	0.047	1.58
3-Hydroxybutyric acid	C ${}_{4}$ H ${}_{8}$ O ${}_{3}$	1.60	0.58	0.048	1.83
n -Butyl lactate	C ${}_{7}$ H ${}_{14}$ O ${}_{3}$	7.68	0.68	0.049	1.21
12-Hydroxydodecanoic acid	C ${}_{12}$ H ${}_{24}$ O ${}_{3}$	12.70	0.59	0.049	1.46
Avasimibe	C ${}_{29}$ H ${}_{43}$ N O ${}_{4}$ S	12.82	0.15	0.049	3.44

${}^{a}$ FC: fold change for the treatment group to control; if the FC value is less than 1, it means that the metabolites were lesser in the treatment group than those in the control group. ${}^{b}$ VIP: variable importance in the projection.

## 4 Discussion

Recently, miRNAs have been shown to regulate gene expression and be involved in the proliferation and differentiation of skeletal muscle (Wang, 2013). Previous evidence has indicated that miR-499-5p regulated skeletal myofiber specification by targeting Sox6 (Nachtigall et al., 2015; Wang et al., 2017), Rod1 (Nachtigall et al., 2015), Thrap1 (Xu et al., 2018), and TGF β R1 (Wu et al., 2019). It was found in our previous study that miR-499-5p levels in skeletal muscle were decreased accompanied by increasing age. The present study demonstrated that miR-499-5p injection significantly decreased the diameter of muscle fiber. The result was consistent with the previous studies because the diameter of slow-twitch muscle fiber was smaller than fast-twitch muscle fiber, and miR-499-5p could regulate skeletal myofiber specification (Xu et al., 2018; Wang et al., 2017; Nachtigall et al., 2015; Wu et al., 2019). However, little is known about the metabolic change of miR-499-5p involvement in the process.

To gain better insight into the significant changes caused by the miR-499-5p injection, we developed an UHPLC-MS/MS method to analyze the endogenous metabolites in broiler muscle. To our knowledge, this is the first study to systematically identify metabolites that are expressed differentially in the muscle of broilers that have been injected by miR-499-5p. The results of PCA and OPLS-DA indicated that there were significant differences in the muscle metabolites of the TG and CG and the levels of 76 metabolites were altered by miR-499-5p, many of which are involved in pathways for metabolizing carbohydrates and lipids.

**Table 3:** Annotation of differential metabolites between the agomiR-499-treated group and the control.

Pathway name	Differential metabolites	P value
Fructose and mannose metabolism	glyceraldehyde 3-phosphate	0.04
Galactose metabolism	glyceraldehyde 3-phosphate	0.04
Inositol phosphate metabolism	glyceraldehyde 3-phosphate	0.04
Terpenoid backbone biosynthesis	glyceraldehyde 3-phosphate	0.04
Glycolysis/gluconeogenesis	glyceraldehyde 3-phosphate	0.08
Caffeine metabolism	xanthine	0.08
Vitamin B6 metabolism	glyceraldehyde 3-phosphate	0.08
Primary bile acid biosynthesis	taurochenodeoxycholic acid	0.12
Thiamine metabolism	glyceraldehyde 3-phosphate	0.12
Pentose phosphate pathway	glyceraldehyde 3-phosphate	0.16
Fatty acid biosynthesis	lauric acid	0.16
Biosynthesis of unsaturated fatty acids	erucic acid	0.20
Purine metabolism	xanthine	0.37

It was shown that the accumulation of lipids in non-adipose tissues elevates the cellular levels of bioactive lipids that inhibit the signaling pathways implicated in metabolic regulation together with an activated inflammatory response (Kang et al., 2013). Specifically, sterol lipids have been shown to influence the fluidity and permeability of membranes (Haines, 2001; Emter et al., 2002) and produce different signaling molecules such as sterol-derived hormones; other sterol-derived signaling molecules include Vitamin D, bile acids, and oxysterols (Hannich et al., 2011; Kurzchalia and Ward, 2003). It may therefore be that the elevated levels of 7alpha-hydroxy-3-oxochol-4-en-24-oic acid and 5-alpha-cholane-3-alpha,7-alpha,12-alpha,24-tetrol in the TG may be beneficial for the functions mentioned above. However, taurochenodeoxycholic acid, as a sterol, was annotated to the pathway of primary bile acid biosynthesis. As a consequence, there might be potential disadvantages of certain functions and the metabolism of host cells responded to miR-499-5p in consideration of the decreased levels of taurochenodeoxycholic acid, about which further research remains to be conducted. Carnitine is a conditionally essential nutrient that acts as an essential factor in fatty acid oxidation in mammals and performs the metabolic function of transporting activated fatty acids into the mitochondria of muscle cells, including those in the heart, for oxidation. It was indicated that miR-499-5p regulates mitochondrial dynamics by targeting calcineurin and dynamin-related protein-1 (J. X. Wang et al., 2011a). Carnitine binds fatty acids, generating various acyl-carnitines with different chain lengths (Flanagan et al., 2010). As shown in Table 2, the levels of 11 long-chain ( ≥ 10 carbons) acyl carnitines were all found to be elevated in the TG. These changes indicated there were different patterns in fatty acid oxidation between the two groups. The muscle is one of the most active tissues for fatty acid oxidation, mainly by the catabolic process of β oxidation. Fatty acid molecules are broken by the process of β oxidation in the mitochondria to generate acetyl coenzyme A (acetyl-coA). Long-chain acyl-carnitines were produced by the reaction of long-chain fatty acyl-CoA and carnitine after long-chain fatty acids were first bound to CoA, and then long-chain acyl-carnitines could be transported across the inner mitochondrial membrane (Luan et al., 2014). The decreased levels of three long-chain fatty acids may be closely associated with increased consumption of long-chain acyl-carnitines in skeletal muscle. Carnitine palmitoyltransferase (CPT) deficiencies are common disorders of mitochondrial fatty acid oxidation (Bonnefont et al., 1999). It is indicated that the inhibition of CPT1 activity was sufficient to substantially diminish food intake and endogenous glucose production (Obici et al., 2003). This is under the unique sensitivity of the outer membrane CPT 1 to the simple molecule, malonyl-CoA (Mcgarry and Brown, 1997). Increased consumption of long-chain acyl-carnitines in muscle may have a relationship with food intake and endogenous glucose production.

Glyceraldehyde 3-phosphate (GAP) is an essential intermediate metabolite in several central pathways of all organisms. GAP can be reversely catalyzed by glyceraldehyde-3-phosphate dehydrogenase (GADPH) into nicotinamide adenine dinucleotide (NADH) and 1,3-bisphosphoglycerate. The increased GAP levels in the TG evidenced the activation of fructose and mannose metabolism, galactose metabolism, inositol phosphate metabolism, and terpenoid backbone biosynthesis in response to the miR-499-5p injection. NADH is a ubiquitous biological molecule that participates in many metabolic reactions in cellular metabolism and energy production. Recent studies showed that NADH played important roles in transcriptional regulation, longevity, calorie-restriction-mediated life span extension, and age-associated diseases (Belenky et al., 2007; Lin and Guarente, 2003; Imai and Guarente, 2014; Verdin, 2015). Collectively, considering the influential roles of GAP within the body, we speculated that the activation of fructose and mannose metabolism, galactose metabolism, inositol phosphate metabolism, and terpenoid backbone biosynthesis could be, at least partially, responsible for the effects of miR-499-5p.

## 5 Conclusions

In summary, miR-499-5p injection resulted in a dramatic decrease in the diameter of muscle fiber. Metabolomics analysis revealed substantial changes in the skeletal muscle metabolite profiles of broilers in response to the miR-499-5p injection. The differential metabolites induced by miR-499-5p were predominantly connected with lipid and carbohydrate metabolism. The results of our study uncovered the complex metabolic effects of miR-499-5p injection, which elucidate fructose and mannose metabolism, galactose metabolism, inositol phosphate metabolism, and terpenoid backbone biosynthesis associated with miR-499-5p, offering new insight into the effect of miR-499-5p on the growth performance of broilers.

## Data Availability

The original data are available upon request to the corresponding author.
